# Multi-Omics of Familial Thoracic Aortic Aneurysm and Dissection: Calcium Transport Impairment Predisposes Aortas to Dissection

**DOI:** 10.3390/ijms242015213

**Published:** 2023-10-16

**Authors:** Shota Tomida, Tamaki Ishima, Daigo Sawaki, Yasushi Imai, Ryozo Nagai, Kenichi Aizawa

**Affiliations:** 1 Division of Clinical Pharmacology, Department of Pharmacology, Jichi Medical University, Shimotsuke 329-0498, Tochigi, Japan; stomida@jichi.ac.jp (S.T.); ishima.tamaki@jichi.ac.jp (T.I.); sawaki@jichi.ac.jp (D.S.); imaiy@jichi.ac.jp (Y.I.); 2 Jichi Medical University, Shimotsuke 329-0498, Tochigi, Japan; rnagai@jichi.ac.jp; 3 Clinical Pharmacology Center, Jichi Medical University Hospital, Shimotsuke 329-0498, Tochigi, Japan; 4 Division of Translational Research, Clinical Research Center, Jichi Medical University Hospital, Shimotsuke 329-0498, Tochigi, Japan

**Keywords:** multi-omics, familial thoracic aortic aneurysm and dissection, calcium transport

## Abstract

Several genetic defects, including a mutation in myosin heavy chain 11 (*Myh11*), are reported to cause familial thoracic aortic aneurysm and dissection (FTAAD). We recently showed that mice lacking K1256 of Myh11 developed aortic dissection when stimulated with angiotensin II, despite the absence of major pathological phenotypic abnormalities prior to stimulation. In this study, we used a comprehensive, data-driven, unbiased, multi-omics approach to find underlying changes in transcription and metabolism that predispose the aorta to dissection in mice harboring the Myh11 K1256del mutation. Pathway analysis of transcriptomes showed that genes involved in membrane transport were downregulated in homozygous mutant (*Myh11*^ΔK/ΔK^) aortas. Furthermore, expanding the analysis with metabolomics showed that two mechanisms that raise the cytosolic Ca^2+^ concentration—multiple calcium channel expression and ADP–ribose synthesis—were attenuated in *Myh11*^ΔK/ΔK^ aortas. We suggest that the impairment of the Ca^2+^ influx attenuates aortic contraction and that suboptimal contraction predisposes the aorta to dissection.

## 1. Introduction

Familial thoracic aortic aneurysm and dissection (FTAAD) accounts for at least 20% of thoracic aortic aneurysms and dissections (TAAD) [1]. Patients with heritable syndromic disease, such as Marfan syndrome and Loeys–Dietz syndrome, are at high risk of developing TAAD [2]. Even though FTAAD is a heritable disease affecting first-degree relatives, patients often do not present syndromic features associated with a heritable syndromic disease [2,3,4,5].

Previously, in two unrelated families, we identified the deletion of Lysine-1256 (K1256del) of MYH11, which encodes myosin heavy chain 11 (Myh11) [6]. We then developed a murine model of FTAAD that possesses K1256del of Myh11 (where heterogeneous and homogeneous mutations are denoted as *Myh11*^ΔK/+^ and *Myh11*^ΔK/ΔK^, respectively) [7]. Although the contractility of *Myh11*^ΔK/+^ aortas was preserved, *Myh11*^ΔK/ΔK^ aortas exhibited a contractile dysfunction [7]. The angiotensin II stimulation of either genotype induced aortic dissection within two weeks, even though aortas from littermate wild-type mice did not develop an aortic dissection after angiotensin II stimulation [7]. This prompted us to hypothesize that there are transcriptomic and metabolic differences even before angiotensin II stimulation that make affected mice more susceptible to aortic dissection. Clinically, the onset of FTAAD does not involve the elevation of angiotensin II, and our ultimate goal is to find transcriptomic and metabolomic abnormalities that can be targeted for treatment and/or diagnosis. Therefore, this study focused on transcriptomic and metabolic abnormalities present without angiotensin II stimulation.

Recently, comprehensive, high-throughput genomics, transcriptomics, proteomics, and metabolomics have contributed greatly to biomedical studies [8]. However, single “omics” studies often only provide correlations [9]. In order to identify the changes that cause disease or prevent recovery from a disease, multi-omics approaches incorporating two or more types of omics data are needed [9]. Various cardiology studies have already integrated multi-omics approaches to understand mechanisms of regeneration and diseases such as coronary artery disease, heart failure, carotid atherosclerosis, aortic valve disease, stroke, and myocardial infarction [10]. The present study takes a comprehensive, data-driven, unbiased approach, combining transcriptomics and metabolomics to further explore the conditions that predispose bearers of Myh11 K1256del to aortic dissection.

## 2. Results

### 2.1. Expression of Genes, Mutations of Which Increase the Risk of Aortic Dissection, Is Unaffected by Myh11 K1256 Deletion

In humans, 10 genes, in addition to *Myh11*, are also involved in the pathogenesis of acute aortic dissection [11]. To determine whether *Myh11*^ΔK/ΔK^ mice share the same mechanisms of aortic dissection as mice harboring those 10 mutations, we first compared the expression of these genes. None of the genes were significantly downregulated in *Myh11*^ΔK/ΔK^ aortas (Supplementary Table S1). Adjusted *p*-values of *Col3a1*, *Smad3*, *Tgfb2*, *Tgfbr1*, or *Tgfbr2* expression were higher than 0.1. *Acta2*, *Fbn1*, *Lox*, *Prkg1,* and *Mylk* were all upregulated (log_2_ fold change = 0.27, 0.23, 1.21, 1.16, and 1.08, respectively). Furthermore, we checked whether the predisposition to aortic dissection could be explained by malfunctioning elastin contractile units. Mutations in genes that contribute to the elastin contractile unit have also been associated with aortic dissection. Those genes include *Efemp2*, *Eln*, *Emilin1*, *Flna*, and *Mfap5*, in addition to *Acta2*, *Fbn1*, *Lox*, *Prkg1,* and *Mylk* [12,13]. However, the expression of these genes in *Myh11*^ΔK/ΔK^ aortas was not significantly lower than that in wild-type aortas (Supplementary Table S2), indicating that a predisposition to aortic dissection in mice harboring the Myh11 K1256 deletion mutation involves a pathway not previously recognized as associated with FTAAD.

### 2.2. Myh11 K1256 Deletion Downregulates Transmembrane Transporters

**Figure 1:**
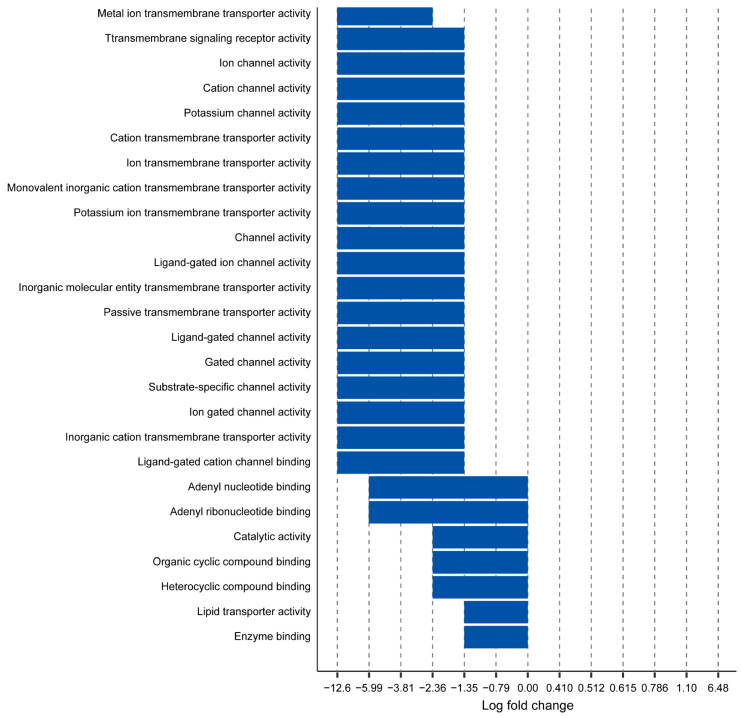
Fold-change-specific enrichment analysis of genes expressed in *Myh11*^∆K/+^ aortas. Names of GO pathways (*y*-axis) are plotted against the range of fold-change values in which they were most significantly enriched (*x*-axis). Negative values indicate downregulation of pathways.

**Figure 2:**
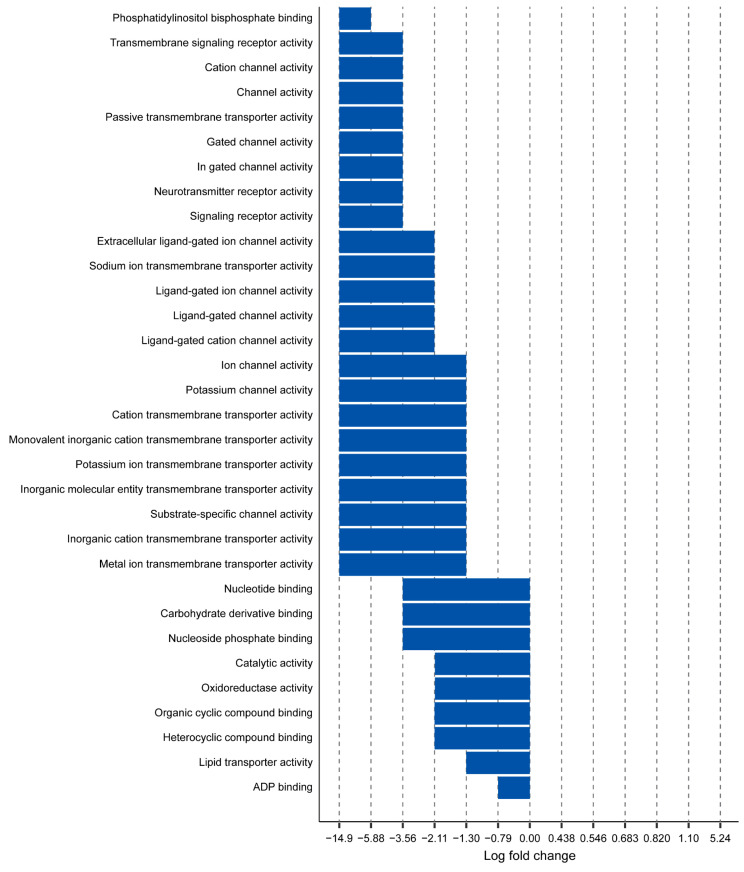
Fold-change-specific enrichment analysis of genes expressed in *Myh11*^∆K/∆K^ aortas. Names of GO pathways (*y*-axis) are plotted against the range of fold-change values in which they were most significantly enriched (*x*-axis). A negative log fold change indicates downregulation of pathways.

**Figure 3:**
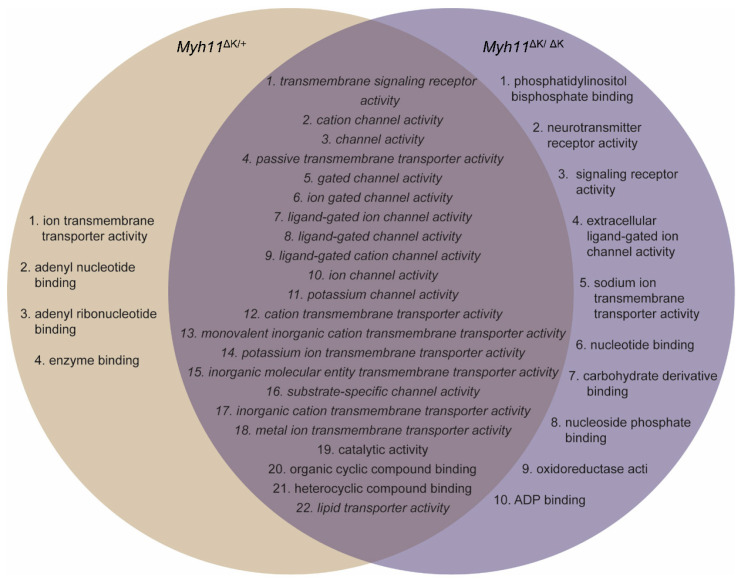
Downregulated GO pathways in *Myh11*^∆K/+^ and *Myh11*^∆K/∆K^ aortas. This Venn diagram shows GO pathways downregulated in both *Myh11*^∆K/+^ and *Myh11*^∆K/∆K^ aortas. Italicized fonts indicate GO pathways related to transmembrane transport.

Using transcriptomic data that we previously obtained of aortas from mice harboring the Myh11 K1256 deletion mutation (denoted *Myh11*^∆K/+^ for heterozygous deletion and *Myh11*^∆K/∆K^ for homozygous deletion) [7], we analyzed genes for which adjusted *p*-values were lower than 0.1, using fold-change-specific enrichment analysis to quantify the degree of change in expression. We found that 26 and 32 molecular functions were attenuated in *Myh11*^∆K/+^ aortas and *Myh11*^∆K/∆K^ aortas, respectively. *Myh11*^∆K/+^ aortas and *Myh11*^∆K/∆K^ aortas share 22 attenuated molecular functions, and none had augmented molecular functions (Figure 1 and Figure 2). Among those 22 molecular functions, 19 were related to transmembrane transporters (Figure 3). In contrast to previous reports on Myh11 mutant mice [14,15], inflammatory response and matrix metalloproteinase expression were not increased (Supplementary Table S3). *Sptbn2* and *Sptbn4*, which encode the non-erythrocytic spectrins that maintain plasma membrane integrity, were downregulated in *Myh11*^∆K/∆K^ (Supplementary Table S4) [16].

### 2.3. Myh11 K1256 Deletion Downregulates Inward Calcium Channels

In a previous study, we found that the contraction of *Myh11*^∆K/∆K^ aortas was attenuated [7]. When searching for transcriptomic abnormalities that might be responsible for attenuated contractions, we investigated genes that contribute to the 22 attenuated molecular functions we identified. We found that calcium channels that transport Ca^2+^ into the cytosol, such as T-type voltage-gated calcium channels (*Cacna1h*: *Myh11*^ΔK/ΔK^/wild-type ratio (log_2_) = −1.20) and nonselective cation channels (*Hcn2, Hcn3, Hcn4, and Tprm2*: log_2_ fold change = −2.50, −5.91, −3.32, and −3.35, respectively), were downregulated (Supplementary Table S5). Conversely, *Myh11*^ΔK/ΔK^ aortas demonstrated a normal expression of plasma membrane Ca^2+^ ATPases (PMCAs), which pump Ca^2+^ out of the cytosol. There was no significant difference between wild-type and *Myh11*^ΔK/ΔK^ aortas in the expression of genes that control Ca^2+^ concentration in the cytosol via Ca^2+^ storage in the sarcoplasmic reticulum, including Sarco-/endoplasmic reticulum Ca^2+^-ATPase, calcium release-activated calcium modulators, stromal interaction molecules, inositol trisphosphate receptors, and ryanodine receptors 1 and 3 (Supplementary Table S6). These expression data suggest that Ca^2+^ influx to the cytosol is attenuated in *Myh11*^ΔK/ΔK^ aortas, but the ejection of Ca^2+^ is as active as wild-type aortas.

### 2.4. Attenuated ADP-Ribose (ADPR) Synthesis in Myh11^ΔK/ΔK^ Aortas Leads to Reduced Cytosolic Ca^2+^

**Figure 4:**
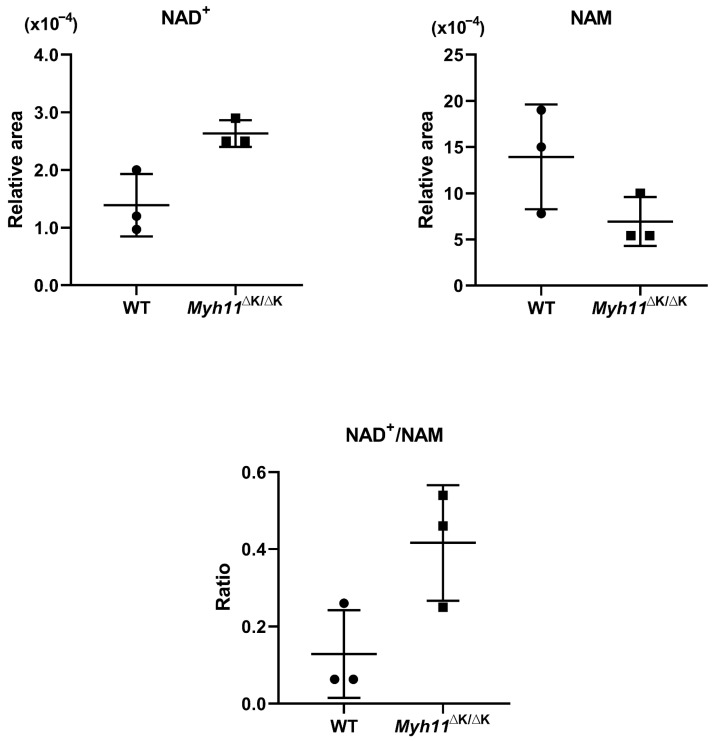
Levels of metabolites related to ADP-ribose synthesis. Scatter plots show mean relative levels ± SD of nicotinamide adenine dinucleotide (NAD^+^) and nicotinamide (NAM), as well as the mean ± SD of the NAD^+^/NAM ratio. n = 3.

Next, we examined transcriptomic abnormalities that enhance Ca^2+^ insufficiency in the cytosol. ADP-ribose stimulates the transient receptor potential cation channel subfamily M member 2 (Trpm2) and increases the influx of Ca^2+^ [17]. Cyclic ADPR induces the release of Ca^2+^ from the sarcoplasmic reticulum to the cytosol [18]. “Catalytic activity” is another attenuated molecular function in *Myh11*^ΔK/ΔK^ aortas identified by FSEA (Figure 3). Poly (ADPR) polymerases (Parp) 6 and 16, which are included in “catalytic activity” by the gene ontology database, were downregulated in *Myh11*^ΔK/ΔK^ aortas (*Parp6* and *Parp16* log_2_ fold changes = −0.57 and −1.04, respectively; Supplementary Table S7). In addition, even though *Sarm1,* which encodes sterile alpha and TIR motif-containing 1, was not listed in the catalytic activity category by gene ontology, it was downregulated (log_2_ fold change = −4.13). Similarly to Parps, Sarm1 synthesizes poly(ADPR) from NAD^+^ (oxidized nicotinamide adenine dinucleotide) and also produces cyclic ADPR [19]. When Parps and Sarm1 synthesize poly(ADPR) by degrading NAD^+^, nicotinamide (NAM) is also synthesized. Coinciding with the downregulation of *Parp6*, *Parp16,* and *Sarm1*, less NAM appeared to be produced in *Myh11*^ΔK/ΔK^ aortas based on a higher NAD^+^/NAM ratio compared to wild-type aortas (Figure 4). Nicotinamide mononucleotide adenylyl transferases (NMNATs), enzymes that convert nicotinamide mononucleotide to NAD^+^, were decreased or unchanged, and the expression of *Nampt* converting NAM to NMN in *Myh11*^ΔK/ΔK^ aortas was unchanged compared to that of wild-type aortas. Furthermore, enzymes that convert NAD^+^ to NADH were also not downregulated in *Myh11*^ΔK/ΔK^ aortas (Supplementary Table S8). Thus, a higher NAD^+^/NAM ratio in *Myh11*^ΔK/ΔK^ aortas was likely due to the attenuation of the conversion of NAD^+^ to poly(ADPR) and not to the augmentation of NAD^+^ synthesis, suggesting that Ca^2+^ insufficiency is further exacerbated by attenuated ADPR synthesis.

### 2.5. ATP Synthesis Is Not Attenuated in Myh11^∆K/∆K^ Aortas

**Figure 5:**
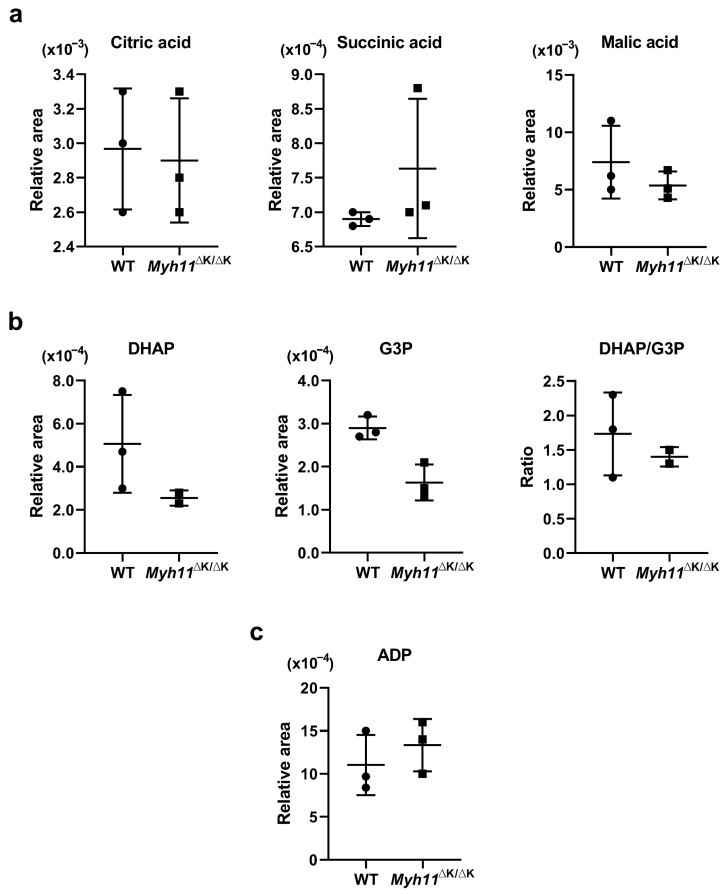
Levels of metabolites involved in the TCA cycle and the electron transport chain. (**a**) Scatter dot plots showing the mean relative levels ± SD of citric acid, succinic acid, and malic acid. (**b**) Scatter dot plots showing the mean relative levels ± SD of dihydroxyacetone phosphate (DAPH) and glycerol-3-phosphate (G3P), as well as the mean ± SD of the DAPH/G3P ratio (n = 3). (**c**) Scatter dot plots showing the mean relative levels ± SD of adenosine diphosphate (ADP) and the mean ± SD of the DAPH/G3P ratio (n = 3).

In addition to smooth muscle contraction, calcium ions also stimulate the tricarboxylic acid (TCA) cycle and oxidative respiration via the activation of various enzymes, yielding a greater amount of ATP [20]. Since phosphate is passed to a myosin light chain from ATP during the contraction of smooth muscle cells [21], we sought to determine whether the expression of enzymes, the levels of metabolites in the TCA cycle, and oxidative respiration in *Myh11*^∆K/∆K^ aortas indicate decreased ATP synthesis. Metabolomic analysis showed that concentrations of TCA cycle intermediates, such as citric acid, succinic acid, and malic acid, in *Myh11*^∆K/∆K^ aortas were similar to those in wild-type aortas (Figure 5a). In *Myh11*^∆K/∆K^ aortas, the expression of genes encoding all enzymes in the TCA cycle, except for *Idh2* (isocitrate dehydrogenase 2), was similar to that in wild-type aortas (Supplementary Table S9). The calcium ion also activates mitochondrial glycerol-3-phosphate dehydrogenase (GPDH), which is encoded by *Gpd2* (wild-type vs. *Myh11*^∆K/∆K^ aortas, adjusted *p*-value = 0.97) [22]. Mitochondrial GPDH converts glycerol-3-phosphate (G3P) to dihydroxyacetone phosphate (DHAP), simultaneously driving mitochondrial respiration by transferring electrons to coenzyme Q [23]. The DHAP:G3P ratio of wild-type and *Myh11*^∆K/∆K^ aortas did not differ (Figure 5b). Furthermore, Myh11 K1256 deletion did not reduce the expression of genes that encode components of the electron transport chain (Supplementary Table S9). In addition, the concentration of ADP in *Myh11*^∆K/∆K^ aortas was comparable to that in wild-type aortas (Figure 5c).

### 2.6. Potassium Channels Are Downregulated in Myh11^ΔK/ΔK^ Aortas

We then analyzed the effect of Myh11 K1256 deletion on the relaxation of smooth muscle. Potassium channels contribute to the relaxation of smooth muscle cells by inducing repolarization and hyperpolarization [24]. In *Myh11*^ΔK/ΔK^ aortas, genes encoding 5 potassium inwardly-rectifying channel subfamily J members were downregulated (*Kcnj3*, *Kcnj9*, *Kcnj10*, *Kcnj12*, and *Kcnj13*: log2 fold change = −3.70, −5.03, −4.61, −2.05, and −6.39, respectively) (Supplementary Table S10), as were 14 voltage-gated potassium channels (Kcna1, Kcna2, Kcna4, Kcna6, Kcnab2, Kcnb2, Kcnc1, Kcnd1, Kcnd2, Kcnh1, Kcnh7, Kcnh8, Kcnip3, and Kcns3 (log2 fold change = −2.03, −2.00, −6.01, −3.52, −3.03, −7.00, −5.04, −1.36, −2.11, −3.74, −7.17, −4.92, −1.07, and −0.57, respectively) (Supplementary Table S11). There was no significant difference in the expression of the α subunit of large-conductance calcium-activated potassium channels (= big potassium channels, BKs) between wild-type and *Myh11*^ΔK/ΔK^ aortas. Even though a β subunit of BK, *Kcnmb1* was slightly upregulated, another subtype of the BK β subunit, *Kcnmb4,* was downregulated in *Myh11*^ΔK/ΔK^ aortas (*Kcnmb1* and *Kcnmb4*: log_2_ fold change = 0.82 and −8.28) (Supplementary Table S12). Three small-conductance calcium-activated potassium channels were downregulated in *Myh11*^ΔK/ΔK^ aortas (*Kcnn2*, *Kcnt1*, and *Kcnu1*: log_2_ fold change = −4.76, −5.64, and −3.72, respectively), and the expression of intermediate conductance calcium-activated potassium channels in wild-type and *Myh11*^ΔK/ΔK^ aortas was comparable (Supplementary Table S12). The downregulation of multiple potassium channels implies the impairment of smooth muscle cell relaxation driven by potassium efflux.

### 2.7. Genes Involved in Nitric Oxide-Induced Smooth Muscle Relaxation Are Upregulated in Myh11^ΔK/ΔK^ Aortas

To further explore the effect of the deletion of K1256 of Myh11 on smooth muscle relaxation, we examined the guanylate cyclase (GC) pathway. One of the mechanisms that triggers smooth muscle cell relaxation is the activation of soluble guanylate cyclase by nitric oxide produced in endothelial cells [25]. The gene expression of nitric oxide synthase 3, which catalyzes arginine conversion to nitric oxide and citrulline in smooth muscle cells, is higher in *Myh11*^ΔK/ΔK^ than in wild-type aortas (log_2_ fold change = 0.53). Furthermore, our metabolomic analysis showed a higher citrulline-to-arginine ratio in *Myh11*^ΔK/ΔK^ than in wild-type aortas, indicating that *Myh11*^ΔK/ΔK^ aortas produce greater amounts of nitric oxide. Even though GCs were not upregulated, both variants of protein kinase G were upregulated in *Myh11*^ΔK/ΔK^ aortas (*Prkg1* and *Prkg2*: log_2_ fold change = 1.16 and 1.14) (Supplementary Table S13), indicating that NO-induced relaxation may be enhanced in *Myh11*^ΔK/ΔK^. 

## 3. Discussion

We analyzed transcriptomic and metabolomic data of aortas from mice harboring the deletion of one lysine residue (K1256) of a quadruple lysine repeat (1253–1256) of Myh11 to identify possible mechanisms of attenuated smooth muscle cell (SMC) contractions leading to aortic dissection.

We first checked whether mice harboring Myh11 K1256 deletion (denoted *Myh11*^ΔK/+^ and *Myh11*^ΔK/ΔK^ for heterozygous and homozygous mutations, respectively) were predisposed to aortic dissection by the same mechanism described in previous reports. In addition to Myh11, mutations in one of 10 other genes predispose humans to acute FTAAD [11]. Furthermore, mutations in the elastin–contractile unit are also associated with FTAAD [12,13]. The transcriptomic analysis did not indicate a significant decrease in the expression of those genes. Thus, the predisposition of *Myh11*^ΔK/ΔK^ mice to FTAAD is likely triggered by a mechanism that has not previously been reported.

To find pathways that are attenuated in both *Myh11*^ΔK/+^ and *Myh11*^ΔK/ΔK^ mice, we ran the fold-change-specific enrichment analysis of transcriptomes, which showed that the Myh11 K1256 deletion mutation mainly downregulates membrane transporters. We identified 22 attenuated molecular functions common in *Myh11*^∆K/+^ and *Myh11*^∆K/∆K^ aortas. Interestingly, 19 of the attenuated molecular functions were transmembrane transporters. Thus, the deletion of one lysine residue of a quadruple lysine repeat of Myh11 likely has a great impact on transmembrane transport.

Calcium membrane transporters take part in attenuated molecular functions, and calcium ions are involved in various stages of SMC contraction [26]. In our previous study, we showed that the contractility of *Myh11*^∆K/∆K^ aortas is weakened. In this study, we found abnormalities in gene expression and metabolome levels that indicate an insufficient cytosolic Ca^2+^ concentration in *Myh11*^∆K/∆K^ aortas. One of the abnormalities involved in the expression of Ca^2+^ membrane transporters is the expression of multiple transmembrane transporters that pass Ca^2+^ into the cytosol, which is lower in *Myh11*^∆K/∆K^ aortas than in wild-type aortas. We propose another mechanism that may cause insufficient cytosolic Ca^2+^ concentration when poly ADP-ribose synthesis is attenuated in *Myh11*^∆K/∆K^ aortas. We found that *Parp6*, *Parp16*, and *Sarm1* in *Myh11*^∆K/∆K^ aortas, which convert NAD^+^ to ADP-ribose and nicotinamide [17], were downregulated, coinciding with higher NAD^+^/NAM ratios compared to wild-type aortas. NAD^+^ accumulation can result from a deficiency in the conversion of NAD^+^ to NADH after the oxidation of NADH during mitochondrial respiration [27]. However, the expression of enzymes that convert NAD^+^ to NADH was not downregulated. This suggests that ADP-ribose synthesis is attenuated in *Myh11*^∆K/∆K^ aortas. Since ADP-ribose increases cytosolic Ca^2+^ concentration, attenuated ADP-ribose synthesis can exacerbate Ca^2+^ insufficiency. We speculate that *Myh11*^∆K/∆K^ SMCs do not demonstrate enough Ca^2+^ binding to calmodulin and that, as a result, calmodulin is unable to activate myosin light chain kinase. It seems likely that the insufficient phosphorylation of the myosin light chain resulting from a decreased cytosolic Ca^2+^ concentration is responsible for the attenuated contraction of *Myh11*^∆K/∆K^.

Another pathway involving the calcium ion is ATP synthesis. Calcium ions drive ATP synthesis by activating the tricarboxylic acid (TCA) cycle and the electron transport chain [20,22]. When smooth muscle cells contract, myosin light chains become phosphorylated by ATP [21]. Thus, we analyzed the transcriptomes and metabolomes of ATP synthetic pathways. The metabolomic analysis did not indicate the inactivation of the TCA cycle in *Myh11*^∆K/∆K^ aortas, as concentrations of intermediates, such as citric acid, succinic acid, malic acid, and gene expression in enzymes of the TCA cycle were normal. In the electron transport chain, calcium ions induce electron transfer to coenzyme Q [23]. During this process, mitochondrial glycerol-3-phosphate dehydrogenase (GPDH) is activated [22], and mitochondrial GPDH converts glycerol-3-phosphate (G3P) to dihydroxyacetone phosphate (DHAP) [23]. We compared the DHAP:G3P ratio between wild-type and *Myh11*^∆K/∆K^ aortas. The DHAP:G3P ratio was not decreased in *Myh11*^∆K/∆K^ aortas; thus, electron transport is likely not attenuated in *Myh11*^∆K/∆K^ aortas. Additionally, although we were not able to detect ATP levels in aortas, the level of ADP in *Myh11*^∆K/∆K^ aortas was not significantly different from that of wild-type aortas. Taken together, metabolomic analysis showed that intermediates of the TCA cycle, electron transport chain, and ADP levels were not attenuated, indicating that ATP synthesis is not suppressed in *Myh11*^∆K/∆K^ aortas.

Another possible cause of attenuated contractility is K^+^ channel downregulation. Since the efflux of K^+^ causes repolarization, the downregulation of K^+^ channels may lead to more positive resting membrane potential. It has been shown that holding membrane potential at −40 mV or −20mV for five seconds lowers L-type Ca^2+^ channel (LTCC) activity by approximately 20% or 60%, respectively [28]. We did not manipulate the membrane potentials of *Myh11*^∆K/∆K^ SMCs, so it is possible that the resting membrane potential was positive enough to partially inactivate LTCCs, attenuating Ca^2+^ influx to the cytosol.

In agreement with our previous study [7], we did not find transcriptomic or metabolomic evidence that smooth muscle relaxation was impaired. Some potassium channel genes were downregulated, which could attenuate relaxation; however, gene expression and metabolite levels suggested that the guanylate cyclase pathway was more active in *Myh11*^∆K/∆K^ aortas than in wild-type aortas. Therefore, we suggest that increased guanylate cyclase activity compensates for the attenuation of relaxation induced by K^+^ and that this mechanism maintains the relaxation of SMCs.

The contractile dysfunction of SMCs has been linked to aortic aneurysm and dissection [29]. Furthermore, an improvement in contraction by rapamycin protects the aortas against aortic dissection in mice who are deficient in type II transforming growth factor-beta receptor expression [30]. However, this contractile dysfunction has been attributed to the disruption of the elastin contractile unit. We showed that the expression of genes previously thought to cause FTAAD is unchanged in *Myh11*^∆K/∆K^ aortas. Thus, a new therapeutic target that improves Ca^2+^ intake in FTAAD patients with Myh11 lysine 1256 deletion could be more effective than a therapy targeting the elastin contractile unit. The T-type calcium channel activator, ZSET1446, has been studied for more than a decade [31,32,33] and has the potential to improve cognition, but it has not been administered to humans. If T-type calcium channel activation improves the contraction of the aorta and ultimately prevents or treats familial aortic dissection, this activator would represent a novel class of aortic dissection medication leading to the development of new drugs.

It has not been reported that Myh11 directly regulates the expression of membrane transporters. Thus, we suggest that there is a cascade of events that leads to the downregulation of membrane transporters. We found that the downregulation of spectrin may link the deletion of one lysine residue to the downregulation of membrane transporters. Spectrins structurally support plasma membranes and connect plasma membranes to the cytosol [16,34]. Two genes encoding spectrin beta chains were downregulated in *Myh11*^∆K/∆K^ aortas. Perhaps *Myh11*^∆K/∆K^ aortas do not have enough spectrin to maintain membrane integrity. Since plasma membrane damage induced by digitonin or fluid shear stress alters expression patterns [35,36], we suggest that decreased plasma membrane integrity triggers the downregulation of membrane proteins, including membrane transporters identified by transcriptome analysis. Furthermore, this may connect the results of the present study to those of our previous one. In that study, we found that the downregulation of integrin in *Myh11*^∆K/∆K^ aortas and smooth muscle cells differentiated from induced pluripotent stem cells [7]. Since integrin is expressed in plasma membranes, we suggest that integrin downregulation is also triggered by the weakening of the plasma membrane, following steps similar to those of membrane transporter downregulation. Additionally, spectrin crosslink networks of short F-actins and the binding of myosin IIA to F-actin contribute to the biconcave shape and deformability of red blood cells [37]. The contractile force of myosin IIA is thought to maintain membrane tension, allowing red blood cells to assume biconcave shapes [37]. Similarly, we suggest that the reduced spectrin expression observed in this study and/or the smooth muscle myosin coiled-coil malformation caused by Myh11 K1256del disrupt the interaction of smooth-muscle myosin and the spectrin-F-actin network resulting in the loss of membrane tension necessary to maintain SMC integrity in our model.

We used two sets of mice for metabolomic and transcriptomic analyses. We believe that transcriptomic and metabolomic data acquired from different sets of mice provide some level of consistency. However, our metabolomic experiment was carried out only once, which could be a limitation of the present study. Repeating the experiment may enhance the credibility of our data.

The comprehensive, data-driven, unbiased approach used in this study revealed a mechanism that hypothesis-driven approaches have overlooked. To the best of our knowledge, this is the first study to connect FTAAD to the impairment of calcium ion uptake by SMCs. We showed that the expression of genes that increase cytosolic Ca^2+^ decreased, which is further supported by metabolomic analysis. Furthermore, the previous study showed that smooth muscle contraction is attenuated in *Myh11*^ΔK/ΔK^ aortas [7]. Even though it has yet to be experimentally confirmed, the present study suggests that cytosolic Ca^2+^ levels are decreased in *Myh11*^ΔK/ΔK^. This may be one mechanism by which Myh11 K1256del predisposes the aorta to dissection. This implies that increasing the cytosolic Ca^2+^ concentration by increasing either poly-ADPR production or Ca^2+^ transporter expression can prevent FTAAD.

## 4. Materials and Methods

### 4.1. Fold-Change-Specific Enrichment Analysis

RNA sequence data were retrieved from a previous publication [7]. According to that study, thoracic aortas were dissected from WT, *Myh11*^∆K/+^, and *Myh11*^∆K/∆K^ mice, and three cDNA samples were prepared from the aortas of each genotype (n = 3) [7]. Genes were first filtered for adjusted *p*-values < 0.1. Using gene names and their fold-change values as inputs, filtered data were analyzed for enrichment using the FoldGO website (https://webfsgor.sysbio.cytogen.ru/run.htm (accessed on 14 September 2021)), which has an algorithm to account for fold changes [38].

### 4.2. Animals

*Myh11* mutant C57BL/6J mice that we previously developed [7] and their littermate wild-type mice were kept under a 12 h light/dark schedule. All animal handling procedures in this study complied with the Jichi Medical University Guide for Laboratory Animals and the ARRIVE guidelines [39]. The Institutional Animal Care and Concern Committee at Jichi Medical University approved all experimental protocols. This study used 12-week-old male mice.

### 4.3. Metabolomic Analysis

Metabolomic analysis was performed on an Agilent CE-TOFMS system. In brief, each aorta was collected and frozen in liquid nitrogen. Samples were homogenized in 450 μL of the extraction solution [(acetonitrile:water = 1:1) with a 10 μM internal standard mixture] at 3500 rpm for 60 s. Homogenization was repeated 4 times. After centrifugation at 2300× *g* and 4 °C for 5 min, supernatants were ultrafiltered into fresh tubes (ultra free MC PLHCC, HMT, centrifugation type filter unit 5 kDa) for centrifugation (9100× *g*, 4 °C, 120 min). Filtered samples were dried and dissolved in 25 μL of water for analysis. Mass spectra were collected in both cation and anion modes of CE-TOFMS with the following settings. Cation mode: sample injection = pressure injection 50 mbar, 10 s; CE voltage = 30 kV; MS ionization = ESI positive; MS capillary voltage = 4000 V; MS scan range = *m*/*z* 50 to 1000. Anion mode: sample injection = pressure injection 50 mbar, 22 s; CE voltage = 30 kV; MS ionization = ESI negative; MS capillary voltage = 3500 V; MS scan range = *m*/*z* 50 to 1000. Acquired peaks were processed using MasterHands automatic integration software ver. 2.9.0.9 (Keio University, Japan). Peaks with signal/noise ratios ≥ 3 were automatically extracted, and mass electric charge ratio (*m*/*z*) areas of peaks alongside migration times were obtained. The relative peak area was calculated by dividing the area of a target peak by the product of the area of the internal standard peak and the sample volume.

### 4.4. Statistical Analysis

*p*-values and adjusted *p*-values of RNA sequence data were calculated using the Wald and Benjamini–Hochberg tests with DeSeq2 [40]. DeSeq2 returns “NA” for log_2_ fold change, *p*-value, and adjusted *p*-value if all samples have zero counts. DeSeq2 returns “NA” for *p*-value and adjusted *p*-value if a dataset contains a sample with an extreme count outlier. DeSeq2 returns “NA” only for the adjusted *p*-value if one or more genotypes (*Myh11*^+/+^ or *Myh11*^ΔK/ΔK^) have a low mean normalized count [41].

## Data Availability

The transcriptomic data presented in this study are openly available in a previous study [7]. Other datasets generated and/or analyzed during the present study are available from the corresponding author on request.

## Supplementary Materials

The following supporting information can be downloaded at: https://www.mdpi.com/article/10.3390/ijms242015213/s1.

## References

[1] Bradley T.J., Bowdin S.C., Morel C.F., Pyeritz R.E. (2016). The Expanding Clinical Spectrum of Extracardiovascular and Cardiovascular Manifestations of Heritable Thoracic Aortic Aneurysm and Dissection. Can. J. Cardiol..

[2] Milewicz D.M., Regalado E.S. (2015). Use of genetics for personalized management of heritable thoracic aortic disease: How do we get there?. J. Thorac. Cardiovasc. Surg..

[3] Biddinger A., Rocklin M., Coselli J., Milewicz D.M. (1997). Familial thoracic aortic dilatations and dissections: A case control study. J. Vasc. Surg..

[4] Coady M.A., Davies R.R., Roberts M., Goldstein L.J., Rogalski M.J., Rizzo J.A., Hammond G.L., Kopf G.S., Elefteriades J.A. (1999). Familial patterns of thoracic aortic aneurysms. Arch. Surg..

[5] Albornoz G., Coady M.A., Roberts M., Davies R.R., Tranquilli M., Rizzo J.A., Elefteriades J.A. (2006). Familial thoracic aortic aneurysms and dissections-incidence, modes of inheritance, and phenotypic patterns. Ann. Thorac. Surg..

[6] Imai Y., Morita H., Takeda N., Miya F., Hyodo H., Fujita D., Tajima T., Tsunoda T., Nagai R., Kubo M. (2015). A deletion mutation in myosin heavy chain 11 causing familial thoracic aortic dissection in two Japanese pedigrees. Int. J. Cardiol..

[7] Negishi K., Aizawa K., Shindo T., Suzuki T., Sakurai T., Saito Y., Miyakawa T., Tanokura M., Kataoka Y., Maeda M. (2022). An Myh11 single lysine deletion causes aortic dissection by reducing aortic structural integrity and contractility. Sci. Rep..

[8] Karczewski K.J., Snyder M.P. (2018). Integrative omics for health and disease. Nat. Rev. Genet..

[9] Hasin Y., Seldin M., Lusis A. (2017). Multi-omics approaches to disease. Genome Biol..

[10] Leon-Mimila P., Wang J., Huertas-Vazquez A. (2019). Relevance of Multi-Omics Studies in Cardiovascular Diseases. Front Cardiovasc. Med..

[11] Pinard A., Jones G.T., Milewicz D.M. (2019). Genetics of Thoracic and Abdominal Aortic Diseases. Circ. Res..

[12] Shen Y.H., Lemaire S.A. (2017). Molecular pathogenesis of genetic and sporadic aortic aneurysms and dissections. Curr. Probl. Surg..

[13] Karimi A., Milewicz D.M. (2016). Structure of the Elastin-Contractile Units in the Thoracic Aorta and How Genes That Cause Thoracic Aortic Aneurysms and Dissections Disrupt This Structure. Can. J. Cardiol..

[14] Pannu H., Tran-Fadulu V., Papke C.L., Scherer S., Liu Y., Presley C., Guo D., Estrera A.L., Safi H.J., Brasier A.R. (2007). MYH11 mutations result in a distinct vascular pathology driven by insulin-like growth factor 1 and angiotensin II. Hum. Mol. Genet..

[15] Chung A.W.Y., Au Yeung K., Sandor G.G.S., Judge D.P., Dietz H.C., Van Breemen C. (2007). Loss of Elastic Fiber Integrity and Reduction of Vascular Smooth Muscle Contraction Resulting From the Upregulated Activities of Matrix Metalloproteinase-2 and -9 in the Thoracic Aortic Aneurysm in Marfan Syndrome. Circ. Res..

[16] Warren M.W., Zheng W., Kobeissy F.H., Cheng Liu M., Hayes R.L., Gold M.S., Larner S.F., Wang K.K. (2007). Calpain- and caspase-mediated alphaII-spectrin and tau proteolysis in rat cerebrocortical neuronal cultures after ecstasy or methamphetamine exposure. Int. J. Neuropsychopharmacol..

[17] Yu P., Cai X., Liang Y., Wang M., Yang W. (2020). Roles of NAD^+^ and Its Metabolites Regulated Calcium Channels in Cancer. Molecules.

[18] Lee H.C. (1997). Mechanisms of calcium signaling by cyclic ADP-ribose and NAADP. Physiol. Rev..

[19] Koch-Nolte F., Fischer S., Haag F., Ziegler M. (2011). Compartmentation of NAD+ -dependent signalling. FEBS Lett..

[20] Brookes P.S., Yoon Y., Robotham J.L., Anders M.W., Sheu S.S. (2004). Calcium, ATP, and ROS: A mitochondrial love-hate triangle. Am. J. Physiol. Cell Physiol..

[21] Deng J.T., Van Lierop J.E., Sutherland C., Walsh M.P. (2001). Ca^2+^-independent Smooth Muscle Contraction. J. Biol. Chem..

[22] McCormack J.G., Denton R.M. (1993). Mitochondrial Ca^2+^ transport and the role of intramitochondrial Ca2+ in the regulation of energy metabolism. Dev. Neurosci..

[23] Mracek T., Drahota Z., Houstek J. (2013). The function and the role of the mitochondrial glycerol-3-phosphate dehydrogenase in mammalian tissues. Biochim. Biophys. Acta.

[24] Nelson M.T., Quayle J.M. (1995). Physiological roles and properties of potassium channels in arterial smooth muscle. Am. J. Physiol..

[25] Denninger J.W., Marletta M.A. (1999). Guanylate cyclase and the ⋅NO/cGMP signaling pathway. Biochim. Biophys. Acta (BBA) Bioenerg..

[26] Wilson D.P., Fitridge R., Thompson M. (2011). Vascular Smooth Muscle Structure and Function. Mechanisms of Vascular Disease: A Reference Book for Vascular Specialists.

[27] Xie N., Zhang L., Gao W., Huang C., Huber P.E., Zhou X., Li C., Shen G., Zou B. (2020). NAD+ metabolism: Pathophysiologic mechanisms and therapeutic potential. Signal Transduct. Target. Ther..

[28] Lacinová L., Hofmann F. (2005). Ca^2+^- and voltage-dependent inactivation of the expressed L-type Cav1.2 calcium channel. Arch. Biochem. Biophys..

[29] Milewicz D.M., Guo D.-C., Tran-Fadulu V., Lafont A.L., Papke C.L., Inamoto S., Kwartler C.S., Pannu H. (2008). Genetic Basis of Thoracic Aortic Aneurysms and Dissections: Focus on Smooth Muscle Cell Contractile Dysfunction. Annu. Rev. Genom. Hum. Genet..

[30] Ferruzzi J., Murtada S.-I., Li G., Jiao Y., Uman S., Ting M.Y.L., Tellides G., Humphrey J.D. (2016). Pharmacologically Improved Contractility Protects Against Aortic Dissection in Mice With Disrupted Transforming Growth Factor-β Signaling Despite Compromised Extracellular Matrix Properties. Arterioscler. Thromb. Vasc. Biol..

[31] Moriguchi S., Shioda N., Yamamoto Y., Tagashira H., Fukunaga K. (2012). The T-type voltage-gated calcium channel as a molecular target of the novel cognitive enhancer ST101: Enhancement of long-term potentiation and CaMKII autophosphorylation in rat cortical slices. J. Neurochem..

[32] Ito Y., Takuma K., Mizoguchi H., Nagai T., Yamada K. (2007). A Novel Azaindolizinone Derivative ZSET1446 (Spiro[imidazo[1,2-a]pyridine-3,2-indan]-2(3H)-one) Improves Methamphetamine-Induced Impairment of Recognition Memory in Mice by Activating Extracellular Signal-Regulated Kinase 1/2. J. Pharmacol. Exp. Ther..

[33] Takeda K., Yamaguchi Y., Hino M., Kato F. (2016). Potentiation of Acetylcholine-Mediated Facilitation of Inhibitory Synaptic Transmission by an Azaindolizione Derivative, ZSET1446 (ST101), in the Rat Hippocampus. J. Pharmacol. Exp. Ther..

[34] De Matteis M.A., Morrow J.S. (2000). Spectrin tethers and mesh in the biosynthetic pathway. J. Cell Sci..

[35] Kunnen S.J., Malas T.B., Semeins C.M., Bakker A.D., Peters D.J.M. (2018). Comprehensive transcriptome analysis of fluid shear stress altered gene expression in renal epithelial cells. J. Cell Physiol..

[36] Häger S.C., Dias C., Sønder S.L., Olsen A.V., da Piedade I., Heitmann A.S.B., Papaleo E., Nylandsted J. (2021). Short-term transcriptomic response to plasma membrane injury. Sci. Rep..

[37] Smith A.S., Nowak R.B., Zhou S., Giannetto M., Gokhin D.S., Papoin J., Ghiran I.C., Blanc L., Wan J., Fowler V.M. (2018). Myosin IIA interacts with the spectrin-actin membrane skeleton to control red blood cell membrane curvature and deformability. Proc Natl. Acad. Sci. USA.

[38] Wiebe D.S., Omelyanchuk N.A., Mukhin A.M., Grosse I., Lashin S.A., Zemlyanskaya E.V., Mironova V.V. (2020). Fold-Change-Specific Enrichment Analysis (FSEA): Quantification of Transcriptional Response Magnitude for Functional Gene Groups. Genes.

[39] Kilkenny C., Browne W., Cuthill I.C., Emerson M., Altman D.G. (2011). Animal Research: Reporting in vivo Experiments—The ARRIVE Guidelines. J. Cereb. Blood Flow Metab..

[40] Love M.I., Huber W., Anders S. (2014). Moderated estimation of fold change and dispersion for RNA-seq data with DESeq2. Genome Biol..

[41] Love M.I., Anders S., Huber W. Analyzing RNA-seq Data with DESeq2. https://bioconductor.org/packages/release/bioc/vignettes/DESeq2/inst/doc/DESeq2.html#pvaluesNA.

