# Supplementary material for: Multi-Omics of Familial Thoracic Aortic Aneurysm and Dissection: Calcium Transport Impairment Predisposes Aortas to Dissection
Source: Int J Mol Sci. 2023 Oct 16;24(20):15213. doi: 10.3390/ijms242015213 (PMC10607035; doi:10.3390/ijms242015213)
Supplement: Supplementary file 1 [file ijms-24-15213-s001.zip › ijms-2639646-supplementary.pdf]

Supplementary information

for

Multi-Omics of Familial Thoracic Aortic Aneurysm and Dissection: Calcium Transport Impairment Predisposes Aortas to Dissection

Shota Tomida [1], Tamaki Ishima [1], Daigo Sawaki [1], Yasushi Imai [1], Ryozo Nagai [2] and Kenichi Aizawa [1,3,4,*]

1 Division of Clinical Pharmacology, Department of Pharmacology, Jichi Medical University, Shimotsuke 329-0498, Tochigi, Japan; stomida@jichi.ac.jp (S.T.); ishima.tamaki@jichi.ac.jp (T.I.); sawaki@jichi.ac.jp (D.S.); imaiy@jichi.ac.jp (Y.I.)
2 Jichi Medical University, Shimotsuke 329-0498, Tochigi, Japan; rnagai@jichi.ac.jp
3 Clinical Pharmacology Center, Jichi Medical University Hospital, Shimotsuke 329-0498, Tochigi, Japan
4 Division of Translational Research, Clinical Research Center, Jichi Medical University Hospital, Shimotsuke 329-0498, Tochigi, Japan
* Correspondence: aizawa@jichi.ac.jp; Tel.: +81-(0)285-58-7388

Supplementary Table S1. Relative expression of genes in *Myh11*$^{\Delta K/\Delta K}$ aortas, deletion of which predisposes mice to FTAAD. $\log_2$ fold change = $\log_2$ of relative expression normalized by expression in wild type aortas. Adjusted p-value = p-values adjusted for multiple testing, determined by the Benjamini and Hochberg method.

**Gene**	**$\log_2$ fold change**	**p-value**	**adjusted p-value**
Acta2	1.03	1.22E−4	0.002
Col3a1	0.38	0.155	0.434
Fbn1	1.00	1.89E−5	0.001
Lox	1.21	2.23E−8	2.52E−6
Mylk	1.08	9.46E−5	0.002
Prkg1	1.16	3.21E−9	5.36E−7
Smad3	0.15	0.398	0.700
Tgfb2	−0.26	0.417	0.714
Tgfbr1	0.52	0.022	0.128
Tgfbr2	0.08	0.601	0.833
Efemp2	0.32	0.213	0.518
Eln	0.63	0.030	0.155
Emilin1	0.34	0.127	0.389
Flna	0.65	0.012	0.084
Mfap5	−0.29	0.201	0.501

Supplementary Table S2. Relative expression of genes involved in the tricarboxylic acid cycle in *Myh11*$^{\Delta K/\Delta K}$ aortas. $\log_2$ fold change = $\log_2$ of relative expression normalized by expression in wild type aortas. Adjusted p-value = p-values adjusted for multiple testing, determined by the Benjamini and Hochberg method.

**Gene**	**$\log_2$ fold change**	**p-value**	**adjusted p-value**
Aco1	−0.34	0.309	0.623
Aco2	−0.63	0.101	0.338
Cs	−0.51	0.121	0.377
Dld	−0.20	0.517	0.784
Dlst	−0.48	0.147	0.423
Fh1	−0.37	0.236	0.545
Idh1	−0.60	0.025	0.140
Idh2	−0.67	0.006	0.052
Idh3a	−0.48	0.145	0.420
Idh3b	−0.41	0.179	0.471
Idh3g	−0.37	0.221	0.526
Mdh1	−0.28	0.142	0.413
Mdh1b	−1.56	0.593	NA
Mdh2	−0.30	0.246	0.559
Ogdh	−0.46	0.157	0.437
Sdha	−0.39	0.221	0.526
Sdhaf1	−0.57	0.017	0.105

Sdhaf2	0.08	0.595	0.830
Sdhaf3	−0.25	0.224	0.530
Sdhaf4	−0.21	0.228	0.536
Sdhb	−0.58	0.048	0.213
Sdhc	−0.13	0.561	0.813
Sdhd	−0.47	0.087	0.310
Sucla2	−0.41	0.190	0.486
Suclg1	−0.44	0.148	0.424
Suclg2	0.08	0.708	0.887

Supplementary Table S3. Relative expression of inflammatory cytokine genes in $\mathit{Myh11}^{\Delta K/\Delta K}$ aortas previously shown to increase in FTAAD.[1, 2] $\log_2$ fold change = $\log_2$ of relative expression normalized by expression in wild-type aortas. Adjusted p-value = p-values adjusted for multiple testing, determined by the Benjamini and Hochberg method.

**Gene**	**$\log_2$ fold change**	**p-value**	**adjusted p-value**
Ccl3	0.16	0.909	NA
Ccl4	0.18	0.903	NA
Igf1	−0.52	0.010	0.075
Mmp2	0.05	0.826	0.937
Mmp9	0.30	0.346	0.657

Supplementary Table S4. Relative expression of spectrin genes in $\mathit{Myh11}^{\Delta K/\Delta K}$ aortas. $\log_2$ fold change = $\log_2$ of relative expression normalized by expression in wild-type aortas. Adjusted p-value = p-values adjusted for multiple testing, determined by the Benjamini and Hochberg method.

**Gene**	**$\log_2$ fold change**	**p-value**	**adjusted p-value**
Spta1	1.12	0.470	NA
Sptan1	0.02	0.885	0.961
Sptb	−1.60	0.007	0.058
Sptbn1	−0.20	0.079	0.291
Sptbn2	−7.00	0.000	0.000
Sptbn4	−4.27	0.000	0.003
Sptbn5	0.01	0.989	0.995

Supplementary Table S5. Relative expression of calcium channel genes in $\mathit{Myh11}^{\Delta K/\Delta K}$ aortas. $\log_2$ fold change = $\log_2$ of relative expression normalized by expression in wild-type aortas. Adjusted p-value = p-values adjusted for multiple testing, determined by the Benjamini and Hochberg method.

**Gene**	**$\log_2$ fold change**	**p-value**	**adjusted p-value**
Cacna1a	−0.96	0.007	0.055
Cacna1b	−10.28	2.60E−7	1.72E−5

Cacna1c	0.52	0.034	0.169
Cacna1d	0.56	0.095	0.325
Cacna1e	−5.56	3.02E−6	1.21E−4
Cacna1f	3.28	0.324	NA
Cacna1g	0.35	0.368	0.677
Cacna1h	−1.20	0.007	0.054
Cacna1i	−0.13	0.929	NA
Cacna1s	−0.09	0.976	NA
Cacna2d1	−0.07	0.740	0.902
Cacna2d2	−4.40	2.35E−6	1.00E−4
Cacna2d3	−2.25	1.92E−7	1.37E−5
Cacna2d4	1.14	0.108	0.353
Cacnb1	−1.50	0.008	0.060
Cacnb2	1.44	7.53E−9	1.03E−6
Cacnb3	−0.58	0.086	0.307
Cacnb4	−2.02	1.13E−4	0.002
Cacng1	−1.11	0.721	NA
Cacng2	−8.93	0.022	0.128
Cacng3	−6.50	0.002	0.019
Cacng4	−1.42	0.060	0.247
Cacng5	−6.07	2.93E−7	1.91E−5
Cacng6	NA	NA	NA
Cacng7	0.06	0.798	0.928
Cacng8	−2.93	0.170	0.458
Hcn1	−2.54	0.017	0.105
Hcn2	−2.50	0.000	0.001
Hcn3	−5.91	0.000	0.006
Hcn4	−3.32	0.001	0.014
Trpa1	NA	NA	NA
Trpc1	0.12	0.515	0.783
Trpc2	NA	NA	NA
Trpc3	−0.09	0.871	0.956
Trpc4	−1.03	0.393	0.697
Trpc4ap	−0.07	0.621	0.841
Trpc5	−1.94	0.486	NA
Trpc5os	NA	NA	NA
Trpc6	−2.11	0.029	0.153
Trpc7	−6.91	0.077	0.288
Trpd52l3	NA	NA	NA
Trpm1	0.06	0.976	NA
Trpm2	−3.35	8.57E−5	0.002
Trpm3	−0.57	0.067	0.263
Trpm4	0.16	0.495	0.768
Trpm5	1.30	0.448	NA
Trpm6	−0.21	0.798	0.927

Trpm7	0.24	0.128	0.391
Trpm8	−0.29	0.920	NA
Trps1	0.10	0.547	0.803
Trpt1	−0.29	0.150	0.428
Trpv1	−0.63	0.276	0.588
Trpv2	−0.35	0.152	0.430
Trpv3	−1.62	0.253	0.566
Trpv4	0.69	0.003	0.030
Trpv5	−1.11	0.785	NA
Trpv6	−1.11	0.785	NA

Supplementary Table S6. Relative expression of genes related to Ca^{2+} release from sarcoplasmic reticulum in *Myh11*$^{\Delta K/\Delta K}$ aortas. $\log_2$ fold change = $\log_2$ of relative expression normalized by expression in wild type aortas. Adjusted p-value = p-values adjusted for multiple testing, determined by the Benjamini and Hochberg method.

Gene	$\log_2$ fold change	p-value	adjusted p-value
Atp2a1	1.63	0.236	0.546
Atp2a2	0.52	0.002	0.023
Atp2a3	0.19	0.452	0.741
Atp2b1	0.15	0.315	0.630
Atp2b2	−1.54	1.31E−7	1.02E−5
Atp2b3	−7.10	0.001	0.007
Atp2b4	−0.09	0.575	0.820
Orai1	0.21	0.255	0.568
Orai2	−0.59	0.139	0.409
Orai3	0.36	0.046	0.206
Stim1	0.08	0.583	0.824
Stim2	0.05	0.743	0.903
Itpr1	0.62	0.006	0.051
Itpr2	−0.60	0.081	0.296
Itpr3	−0.10	0.575	0.820

Supplementary Table S7. Relative expression of ADP-ribose polymerase genes in *Myh11*$^{\Delta K/\Delta K}$ aortas. $\log_2$ fold change = $\log_2$ of relative expression normalized by expression in wild type aortas. Adjusted p-value = p-values adjusted for multiple testing, determined by the Benjamini and Hochberg method.

Gene	$\log_2$ fold change	p-value	adjusted p-value
Parp1	−0.11	0.445	0.737
Parp2	0.18	0.330	0.643
Parp3	0.10	0.564	0.814
Parp4	−0.36	0.043	0.200
Parp6	−0.57	0.005	0.042

Parp8	0.32	0.129	0.391
Parp9	−0.14	0.360	0.671
Parp10	−0.50	0.031	0.160
Parp11	−0.50	0.157	0.437
Parp12	−0.12	0.452	0.741
Parp14	−0.27	0.152	0.431
Parp16	−1.04	0.001	0.007
Tiparp	−0.51	0.036	0.177
Sirt1	0.22	0.187	0.482
Sirt2	−0.09	0.383	0.688
Sirt3	−0.30	0.135	0.402
Sirt4	0.16	0.617	0.840
Sirt5	−0.26	0.268	0.581
Sirt6	−0.06	0.798	0.927
Sirt7	−0.11	0.488	0.764
CD38	−0.48	0.138	0.407
Sarm1	−4.13	9.66E−8	8.13E−6
Nmnat1	−0.13	0.600	0.833
Nmnat2	−3.44	0.000	0.007
Nmnat3	−0.82	0.026	0.141
Nampt	-0.23	0.323	0.637

Supplementary Table S8. Relative expression of genes that convert NAD^+ to NADH in $Myh11^{\Delta K/\Delta K}$ aortas. $\log_2$ fold change = $\log_2$ of relative expression normalized by expression in wild type aortas. Adjusted p-value = p-values adjusted for multiple testing, determined by the Benjamini and Hochberg method.

**Gene**	**$\log_2$ fold change**	**p-value**	**adjusted p-value**
Dlat	-0.16	0.593	0.829
Dld	-0.20	0.517	0.784
Dlst	-0.48	0.147	0.423
Gapdh	0.30	0.577	0.821
Idh2	-0.67	0.006	0.052
Mdh1	-0.28	0.142	0.413
Mdh1b	-1.56	0.593	NA
Mdh2	-0.30	0.246	0.559
Ogdh	-0.46	0.157	0.437
Pdha1	-0.03	0.915	0.971
Pdha2	NA	NA	NA
Pdhb	0.24	0.427	0.724

Supplementary Table S9. Relative expression of genes involved in the electron transport chain in $Myh11^{\Delta K/\Delta K}$ aortas. $\log_2$ fold change = $\log_2$ of relative expression normalized by expression in wild type aortas. Adjusted p-value = p-values adjusted for multiple testing, determined by the Benjamini and Hochberg method.

Gene	log_2 fold change	p-value	adjusted p-value
Bcs1l	−0.25	0.319	0.633
Coa3	−0.04	0.832	0.940
Coa4	−0.45	0.249	0.562
Coa5	−0.25	0.337	0.649
Coa6	0.09	0.645	0.854
Coa7	−0.31	0.267	0.580
Cox10	−0.61	0.034	0.171
Cox11	0.09	0.641	0.852
Cox14	−0.31	0.059	0.245
Cox15	−0.22	0.281	0.594
Cox16	0.10	0.770	0.917
Cox17	−0.26	0.265	0.577
Cox18	−0.21	0.356	0.667
Cox19	0.19	0.348	0.659
Cox20	0.08	0.547	0.803
Cox4i1	−0.43	0.060	0.246
Cox4i2	0.27	0.398	0.700
Cox5a	−0.54	0.074	0.280
Cox5b	−0.29	0.275	0.588
Cox6a1	−0.24	0.299	0.613
Cox6a2	2.07	0.091	0.318
Cox6b1	−0.46	0.076	0.285
Cox6b2	−0.24	0.549	0.804
Cox6c	−0.08	0.677	0.872
Cox7a1	−0.74	0.053	0.227
Cox7a2	−0.16	0.342	0.653
Cox7a2l	0.00	0.978	0.992
Cox7b	−0.16	0.483	0.761
Cox7b2	−1.11	0.785	NA
Cox7c	0.14	0.794	0.926
Cox8a	−0.26	0.256	0.569
Cox8b	−0.63	0.095	0.327
Cox8c	NA	NA	NA
Cyc1	−0.47	0.114	0.364
mt-Co1	−0.38	0.073	0.279
mt-Co2	NA	NA	NA
mt-Co3	−0.15	0.970	NA
mt-Cytb	−0.31	0.146	0.420
Ndufa1	−0.16	0.434	0.729
Ndufa10	−0.44	0.166	0.451
Ndufa11	−0.02	0.895	0.965
Ndufa12	−0.21	0.284	0.597
Ndufa13	−0.03	0.864	0.954
Ndufa2	0.11	0.379	0.684

Ndufa3	−0.24	0.263	0.576
Ndufa4	−0.17	0.406	0.706
Ndufa4l2	0.06	0.821	0.935
Ndufa5	−0.19	0.304	0.619
Ndufa6	−0.04	0.785	0.923
Ndufa7	−0.19	0.256	0.569
Ndufa8	−0.36	0.102	0.342
Ndufa9	−0.42	0.116	0.368
Ndufab1	−0.11	0.751	0.907
Ndufab1-ps	−1.11	0.785	NA
Ndufaf1	−0.42	0.060	0.246
Ndufaf2	0.08	0.695	0.881
Ndufaf3	−0.04	0.820	0.935
Ndufaf4	−0.08	0.631	0.847
Ndufaf5	−0.20	0.257	0.569
Ndufaf6	−0.26	0.355	0.666
Ndufaf7	−0.14	0.351	0.662
Ndufaf8	0.09	0.598	0.832
Ndufb10	−0.38	0.065	0.258
Ndufb11	−0.18	0.359	0.670
Ndufb2	0.04	0.798	0.927
Ndufb3	−0.03	0.869	0.955
Ndufb4	−0.28	0.204	0.505
Ndufb5	−0.25	0.250	0.563
Ndufb6	−0.25	0.319	0.633
Ndufb7	−0.20	0.218	0.523
Ndufb8	−0.22	0.371	0.679
Ndufb9	−0.32	0.178	0.469
Ndufc1	−0.22	0.271	0.583
Ndufc2	−0.30	0.143	0.415
Ndufs1	−0.29	0.324	0.637
Ndufs2	−0.25	0.385	0.690
Ndufs3	−0.35	0.192	0.489
Ndufs4	−0.09	0.660	0.864
Ndufs5	−0.03	0.917	0.971
Ndufs6	−0.21	0.305	0.619
Ndufs7	−0.51	0.036	0.175
Ndufs8	−0.35	0.154	0.433
Ndufv1	−0.47	0.144	0.419
Ndufv2	−0.29	0.200	0.500
Ndufv3	−0.28	0.160	0.443
Sco1	0.01	0.951	0.983
Sco2	−0.44	0.195	0.492
Sdha	−0.39	0.221	0.526
Sdhaf1	−0.57	0.017	0.105

Sdhaf2	0.08	0.595	0.830
Sdhaf3	−0.25	0.224	0.530
Sdhaf4	−0.21	0.228	0.536
Sdhb	−0.58	0.048	0.213
Sdhc	−0.13	0.561	0.813
Sdhd	−0.47	0.087	0.310
Surf1	−0.09	0.487	0.763
Uqcc1	−0.61	0.012	0.086
Uqcc2	0.03	0.842	0.944
Uqcc3	−0.31	0.109	0.354
Uqcr10	−0.16	0.454	0.742
Uqcr11	−0.26	0.269	0.582
Uqcrb	−0.01	0.956	0.985
Uqcrc1	−0.45	0.136	0.403
Uqcrc2	−0.26	0.365	0.674
Uqcrfs1	−0.33	0.279	0.592
Uqcrh	0.01	0.970	0.989
Uqcrq	−0.18	0.452	0.741

Supplementary Table S10. Relative expression of inwardly rectifying potassium channel genes in *Myh11*$^{\Delta K/\Delta K}$ aortas. $\log_2$ fold change = $\log_2$ of relative expression normalized by expression in wild type aortas. Adjusted p-value = p-values adjusted for multiple testing, determined by the Benjamini and Hochberg method.

Gene	$\log_2$ fold change	p-value	adjusted p-value
Kcnj1	NA	NA	NA
Kcnj2	−0.75	0.169	0.456
Kcnj3	−3.70	0.002	0.018
Kcnj4	0.06	0.959	0.986
Kcnj5	4.29	0.033	NA
Kcnj6	NA	NA	NA
Kcnj8	0.79	0.004	0.035
Kcnj9	−5.03	0.002	0.019
Kcnj10	−4.61	9.20E−07	4.80E−05
Kcnj11	−0.44	0.196	0.493
Kcnj12	−2.05	0.000	0.004
Kcnj13	−6.39	0.000	0.005
Kcnj14	2.15	0.426	NA
Kcnj15	0.32	0.268	0.580
Kcnj16	−6.59	0.091	0.318
Kcna10	NA	NA	NA

Supplementary Table S11. Relative expression of voltage-gated potassium channel genes in *Myh11*$^{\Delta K/\Delta K}$ aortas. $\log_2$ fold change = $\log_2$ of relative expression normalized by expression in

wild type aortas. Adjusted p-value = p-values adjusted for multiple testing, determined by the Benjamini and Hochberg method.

Gene	log_2 fold change	p-value	adjusted p-value
Kcna1	−2.03	2.98E−4	0.005
Kcna2	−2.00	1.05E−4	0.002
Kcna3	−1.29	0.275	0.588
Kcna4	−6.01	8.92E−4	0.011
Kcna5	−0.34	0.354	0.666
Kcna6	−3.52	9.70E−9	1.28E−6
Kcna7	0.78	0.801	NA
Kcna10	NA	NA	NA
Kcnab1	0.91	6.21E−4	0.008
Kcnab2	−3.03	7.90E−06	2.58E−4
Kcnab3	−0.28	0.619	0.840
Kcnb1	0.30	0.191	0.487
Kcnb2	−7.00	6.11E−5	0.001
Kcnc1	−5.04	0.003	0.029
Kcnc2	−0.32	0.642	0.853
Kcnc3	−1.55	0.055	0.232
Kcnc4	−0.58	0.215	0.520
Kcnd1	−1.36	0.008	0.063
Kcnd2	−2.11	0.001	0.016
Kcnd3	0.49	0.072	0.276
Kcne1	NA	NA	NA
Kcne1l	−2.10	0.057	0.237
Kcne2	NA	NA	NA
Kcne3	−0.11	0.847	0.946
Kcne4	0.74	0.012	0.081
Kcnf1	0.91	0.458	0.745
Kcng1	−0.55	0.765	NA
Kcng2	0.15	0.896	0.965
Kcng3	−0.15	0.970	NA
Kcng4	0.40	0.544	0.802
Kcnh1	−3.74	9.91E−8	8.24E−6
Kcnh2	0.11	0.662	0.865
Kcnh3	0.31	0.804	0.930
Kcnh4	0.43	0.677	0.872
Kcnh5	−1.92	0.635	NA
Kcnh6	−7.06	0.016	0.103
Kcnh7	−7.17	4.66E−6	1.69E−4
Kcnh8	−4.92	6.65E−4	0.009
Kcnip1	−0.13	0.959	NA
Kcnip2	−2.66	0.036	0.175
Kcnip3	−1.07	2.42E−4	0.004

Kcnip4	−0.82	0.08	0.31
Kcns1	NA	NA	NA
Kcns2	−6.73	0.085	0.305
Kcns3	−0.57	NA	NA
Kcnv1	−1.11	0.085	0.306
Kcnv2	1.73	0.009	0.067

Supplementary Table S12. Relative expression of voltage-gated large and small conductance calcium-activated potassium channel genes in *Myh11*$^{\Delta K/\Delta K}$ aortas. $\log_2$ fold change = $\log_2$ of relative expression normalized by expression in wild type aortas. Adjusted p-value = p-values adjusted for multiple testing, determined by the Benjamini and Hochberg method.

Gene	$\log_2$ fold change	p-value	adjusted p-value
Kcnma1	0.13	0.587	0.826
Kcnmb1	0.82	0.001	0.009
Kcnmb2	NA	NA	NA
Kcnmb3	NA	NA	NA
Kcnmb4	−8.28	2.53E−9	4.55E−7
Kcnn1	−0.61	0.021	0.122
Kcnn2	−4.76	1.24E−5	3.75E−4
Kcnn3	−0.74	0.035	0.172
Kcnn4	0.03	0.934	0.976
Kcnt1	−5.64	3.48E−6	1.36E−4
Kcnt2	0.18	0.487	0.764
Kcnu1	−3.72	0.009	0.068

Supplementary Table S13. Relative expression of genes involved in protease kinase G (PKG) pathway in *Myh11*$^{\Delta K/\Delta K}$ aortas. $\log_2$ fold change = $\log_2$ of relative expression normalized by expression in wild type aortas. Adjusted p-value = p-values adjusted for multiple testing, determined by the Benjamini and Hochberg method.

Gene	$\log_2$ fold change	p-value	adjusted p-value
Gucy1a2	0.05	0.859	0.952
Gucy1a3	−0.06	0.763	0.912
Gucy1b2	−1.11	0.785	NA
Gucy1b3	0.18	0.342	0.653
Gucy2c	−0.05	0.984	NA
Gucy2d	−1.11	0.785	NA
Gucy2e	−3.74	0.219	NA
Gucy2f	−1.11	0.785	NA
Gucy2g	−0.33	0.791	0.925
Guf1	0.06	0.724	0.894
Guk1	−0.27	0.027	0.145

Prkg1	1.16	3.21E−9	5.36E−7
Prkg2	1.15	0.006	0.053

References

1. Pannu H, *et al.* MYH11 mutations result in a distinct vascular pathology driven by insulin-like growth factor 1 and angiotensin II. *Human Molecular Genetics* **16**, 2453-2462 (2007).

2. Chung AWY, Au Yeung K, Sandor GGS, Judge DP, Dietz HC, Van Breemen C. Loss of Elastic Fiber Integrity and Reduction of Vascular Smooth Muscle Contraction Resulting From the Upregulated Activities of Matrix Metalloproteinase-2 and -9 in the Thoracic Aortic Aneurysm in Marfan Syndrome. *Circulation Research* **101**, 512-522 (2007).
